# Short Faces, Big Tongues: Developmental Origin of the Human Chin

**DOI:** 10.1371/journal.pone.0081287

**Published:** 2013-11-15

**Authors:** Michael Coquerelle, Juan Carlos Prados-Frutos, Rosa Rojo, Philipp Mitteroecker, Markus Bastir

**Affiliations:** 1 Paleoanthropology group, Museo Nacional de Ciencias Naturales (CSIC), Madrid, Spain; 2 Department of Stomatology, University Rey Juan Carlos, Alcorcon, Spain; 3 Department of Theoretical Biology, University of Vienna, Vienna, Austria; University of Kansas, United States of America

## Abstract

During the course of human evolution, the retraction of the face underneath the braincase, and closer to the cervical column, has reduced the horizontal dimension of the vocal tract. By contrast, the relative size of the tongue has not been reduced, implying a rearrangement of the space at the back of the vocal tract to allow breathing and swallowing. This may have left a morphological signature such as a chin (mental prominence) that can potentially be interpreted in *Homo*. Long considered an autopomorphic trait of *Homo sapiens*, various extinct hominins show different forms of mental prominence. These features may be the evolutionary by-product of equivalent developmental constraints correlated with an enlarged tongue. In order to investigate developmental mechanisms related to this hypothesis, we compare modern 34 human infants against 8 chimpanzee fetuses, whom development of the mandibular symphysis passes through similar stages. The study sets out to test that the shared ontogenetic shape changes of the symphysis observed in both species are driven by the same factor – the space restriction at the back of the vocal tract and the associated arrangement of the tongue and hyoid bone. We apply geometric morphometric methods to extensive three-dimensional anatomical landmarks and semilandmarks configuration, capturing the geometry of the cervico-craniofacial complex including the hyoid bone, tongue muscle and the mandible. We demonstrate that in both species, the forward displacement of the mental region derives from the arrangement of the tongue and hyoid bone, in order to cope with the relative horizontal narrowing of the oral cavity. Because humans and chimpanzees share this pattern of developmental integration, the different forms of mental prominence seen in some extinct hominids likely originate from equivalent ontogenetic constraints. Variations in this process could account for similar morphologies.

## Introduction

Numerous specimens of the hominid fossil record are represented by mandibular remains and their taxonomic attributions are often based on interpretations of certain external aspects of the symphyseal morphology, such as the mental region on the labial side of the symphysis. It has long been accepted that a protruding mental region (chin) is an autapomorphic character defining modern humans [1]. However, different forms of protrusion of the mental region have been identified in various extinct hominids other than *Homo sapiens*, for instance the adult Neanderthals Guattari 3, La Quina 9, Saint-Césaire, Vindja 206, as well as the Atapuerca specimens AT605 and AT300 [2-12]. As a result the mental prominence remains a confusing feature from an evolutionary perspective and its taxonomic significance is not clear. Furthermore, the current literature still questions whether these different forms of protrusion can be seen as ontogenetically equivalent morphological features or not. Could the mental prominence have emerged from a common developmental pathway linked to identical changes of the cervico-craniofacial configuration? If this is true, it may imply that the prominence of the mental region has a low taxonomic significance because, throughout human evolution, we and our ancestors faced identical developmental constraints, most importantly the ability to breathe and swallow.

### Evolution

These constraints can be assumed because during the course of human evolution, with the introduction of meat into the diet and the invention of cooking [13-15], dramatic changes occurred to the cranial base, due to brain enlargement, and to the face and teeth, characterized by the reduction of prognathism and size [16-20]. Because the face repositions beneath the anterior cranial base and closer to the cervical vertebrae, the anteroposterior dimension of the vocal tract reduces relatively and absolutely. By contrast, the relative size of the human tongue has not been reduced [20,21]. As in chimpanzees, the human tongue is long and flat at birth, occupying almost the entire mouth, and leaving little space for the airway between the back of the oral cavity and the cervical vertebrae. Why this has been selected during evolution is unclear as well as the selection for the high positioned larynx, which is also associated with the form of the neonatal tongue [21]. Functionally, such an arrangement is presumably related to breastfeeding [20]. But afterwards, during early postnatal growth, how do modern humans cope with a large tongue? Driven by the need to breathe and swallow safely, the prominence of the mental region may result from the need for space at the back of the vocal tract related to the spatial arrangement and size of the tongue. This hypothesis has a long history [22] but has never been explored empirically. Our study sets out to test this hypothesis via comparative early development of the modern human and chimpanzee cervico-craniofacial anatomy.

In extinct hominids with a prominent mental region, mandibular growth may have been associated with spatial constraints similar to those characterizing modern humans. The preservation of the hominid fossil record does not allow this question to be addressed empirically. Nevertheless, investigating pre- and early postnatal ontogeny of humans and chimpanzees helps identify morphological similarities and sources of variation underlying their phylogenetic changes [23-27]. In addition, the crania of African apes and modern humans have similar patterns of developmental integration, implying that the effects of common factors are fairly conserved among hominoids [28-30]. We hypothesize that patterns of developmental integration in the oral cavity are also conserved because all primates experience the same selection pressure to maintain an open oral cavity sufficient for breathing and swallowing. 

### Development

In modern humans and chimpanzees, development of the mandibular symphysis passes through similar stages albeit at different times. This includes the forward shift of the mental region leading to a vertical symphysis with a mental protuberance in chimpanzee fetuses, but a mental prominence in human infants. In addition, within the bone, the deciduous incisors become vertically oriented prior to eruption in both species [31]. In infant modern humans, it has been demonstrated that both the forward projection of the mental region and the incisor reorientation are correlated with the relocation of the tongue and suprahyoid muscle insertions at the lingual side of the mental region [32]. Looking at the symphyseal region alone, this developmental integration accounts for the biomechanical properties of the protrusive mental region during biting [33]. However when the entire cervico-craniofacial complex is taken into consideration, the symphyseal shape changes seem to be a response to the horizontal space restriction at the back of the vocal tract [32]. This space restriction is caused by both the backward positioning of the upper mid-face concomitant with the flexion of the cranial base, and the forward positioning of the cervical column and hyoid bone due to the development of upright posture [32]. In such a developmental context, it is even more striking that the large tongue, with a growth rate following a neural pattern rather than a somatic one such as the face [34,35], needs to fit in the small oral cavity and does so by remodelling its neonatal shape. At the same time, this is likely to alter the position and orientation of the suprahyoid muscles and the hyoid bone located below the tongue while maintaining the ability to breathe and swallow safely.

In neonate chimpanzees, the hyo-laryngeal structures lie at their adult level relative to the cervical vertebrae and the position of the hyoid bone is anterior to the gonial angles, as in modern humans after 2 years [21,36-40]. The forward positioning of the hyoid bone relative to the inferior border of the symphysis seems to be coordinated with the development of a vertical symphysis in chimpanzee fetuses but a mental prominence in human infants [31]. However, such a shared pattern of developmental integration still needs to be demonstrated.

In chimpanzee fetuses, the similar shape changes observed at the symphysis during development occurs in a different environment, the womb. During fetal life of primates the aerodigestive tract is full of amniotic fluids and the pharyngeal and laryngeal functions begins by the end of the first trimester. Fetal breathing of amniotic fluids at a tidal flow and low-frequency movements of the tongue during swallowing contribute to shaping and maintaining the form of the aerodigestive tract [41-44]. In chimpanzee fetuses, the flexion of the head towards the thorax, characterising fetal position, is likely to modify the position of the cervical column, the pharynx and the larynx including the hyoid bone relative to the mandibular symphysis. This may cause spatial adjustments and constraints at the back of the vocal tract, similar to those of infant modern humans though related to different developmental reasons, in order to maintain fetal breathing and swallowing. Concomitantly the position of the tongue and the suprahyoid muscles and the orientation of the muscle forces are likely to change, along with the relocation of the hyoid bone, influencing the direction of bone growth at symphysis. The anatomical changes at the back of the vocal tract have not yet been documented in chimpanzee fetuses and so remain to be explored in relation to the shape changes of the mandibular symphysis.

### Aim of the study

**Figure 1:**
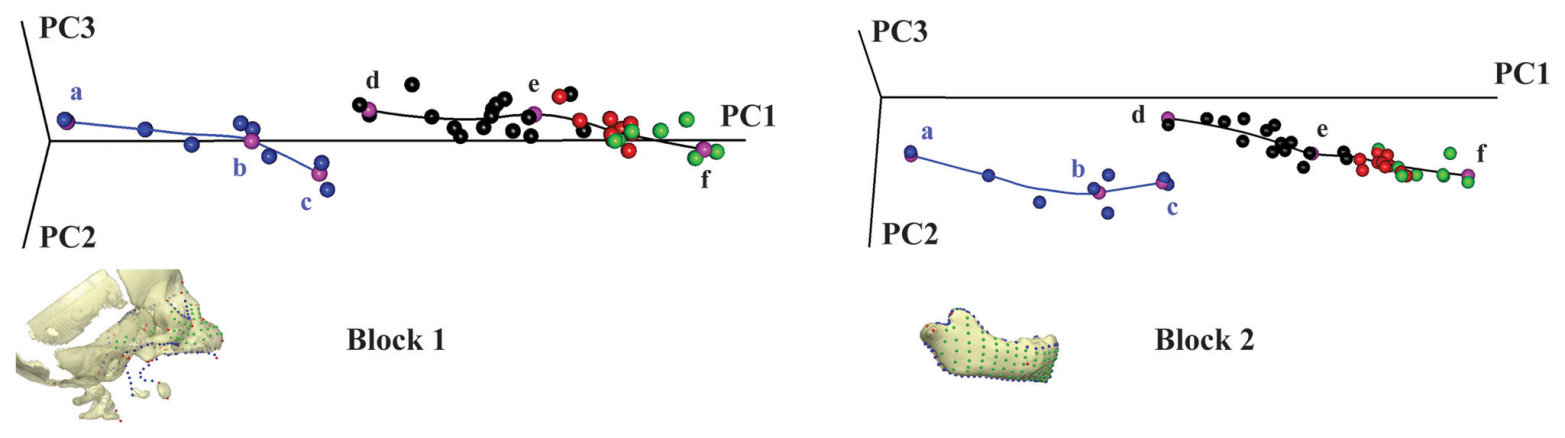
Form space principal component analysis. The first three PCs in Procrustes form space account for approximately 94% of the total form variance for block 1 and 98% of that for block 2. Chimpanzees (blue trajectory) and humans (black trajectory) have distinct curvilinear ontogenetic trajectories. Blue dots: chimpanzee fetuses (a, b) to neonates (c). Black dots: humans from birth (d) to ~1 y.o. (e), red dots: from ~1 to ~2.5 y.o., green dots: from 2.5 to 5.5 y.o (f). Pink dots: regression estimates at age a, b, c, d, e and f visualized in Figure 2. The 3D reconstructions of blocks 1 and 2 show the landmark and semilandmark configurations (see Figure S1 and Table S1 for complete description). Note the parallel orientations of trajectories between a) and b) in chimps and between d) and e) in humans demonstrates similar developmental pathways within a different anatomical context.

We test the hypothesis that the shared developmental changes of the symphysis observed in chimpanzee fetuses and modern human infants [31] are driven by the same factor – the space restriction at the back of the vocal tract and the associated arrangement of the tongue and hyoid bone. To this end, we apply geometric morphometric methods to extensive three-dimensional anatomical landmarks and semilandmarks configuration, capturing the geometry of the cervico-craniofacial complex including the hyoid bone, tongue muscle (Figure 1a) and the mandible (Figure 1b). This represents the first study which thoroughly measures fetal and early early postnatal craniofacial morphogenesis in 3D, including hard and soft tissue, and providing original data on cervico-craniofacial morphology during chimpanzee fetal ontogeny.

## Material and Methods

### Computer Tomography (CT) sample.

The postnatal human sample consists of 34 CT scanned modern humans (15 females, 19 males; age range: from birth to approximately 5.5 years of age) of mixed ethnicity from France. The CT scans were provided by the Pellegrin Hospital (Bordeaux, n = 22), the Necker Hospital (Paris, n = 4), and the Clinic Pasteur (Toulouse, n = 8). This human sample is a subset of the sample used in previous studies on mandibular growth [31,32,45]. The human specimens had been referred for cranial trauma but were found to be free of reportable abnormalities. Prior to analysis all CT-data were anonymized to comply with the Helsinki declaration [46]. Because the subjects were scanned previously for medical reasons unrelated to this study (retrospective), it is lawful and not necessary to obtain consent from the next of kin, caretakers, or guardians on the behalf of minors/children participants of this study. Consequently such consent was not required by the local ethic committees following local laws. The approval to use these pre-existing CT scans, gathered from the three medical institutes cited above, for our research was obtained in writing from the *Comité consultatif pour la protection des personnes dans la recherche biomédicale Bordeaux A* (copy of approval of the ethics committee has been submitted to manuscript central).

The chimpanzee sample included 8 CT-scanned formaldehyde fixed *Pan troglodytes* (sex unknown; age range: from the 18^th^ g. w. to birth) provided by the Musée de l’Homme (Paris). Age assessment of the chimpanzees as well as the characteristics of the human and chimpanzee CT scans, and the techniques and software used for the reconstruction the bony surface have been detailed in various studies [31,32,45]. In the modern human sample, although postnatal mandibular growth is sexually dimorphic, sex differences are very small compared to age differences and consist mainly of differences in developmental timing [45]. Therefore, human males and females are not distinguished in this study.

### The 3D shape coordinates

We digitized 686 3D landmarks and semilandmarks to capture the geometry of block 1, which includes the upper mid-face, the basicranium, the cervical column, the hyoid bone and the midline of the tongue; and block 2, composed of the mandible (Figure 1, Figure S1, Table S1). Blocks 1 and 2 (semi)landmarks were digitized separately using the software Viewbox 4 (dHAL Software, Kifissia, Greece). The semilandmarks were allowed to slide along curves and surfaces to minimize the bending energy of the thin-plate spline interpolation function computed between each specimen and the sample Procrustes average [47,48]. After sliding, landmarks and semilandmarks were treated as homologous points and converted to shape coordinates by Generalized Procrustes Analysis [49]. This involves rescaling the landmark coordinates so that each configuration has a unit Centroid Size (CS). Then all configurations are translated and rotated to minimize the overall sum of the squared distances between corresponding (semi)landmarks.

### Analyses

**Figure 2:**
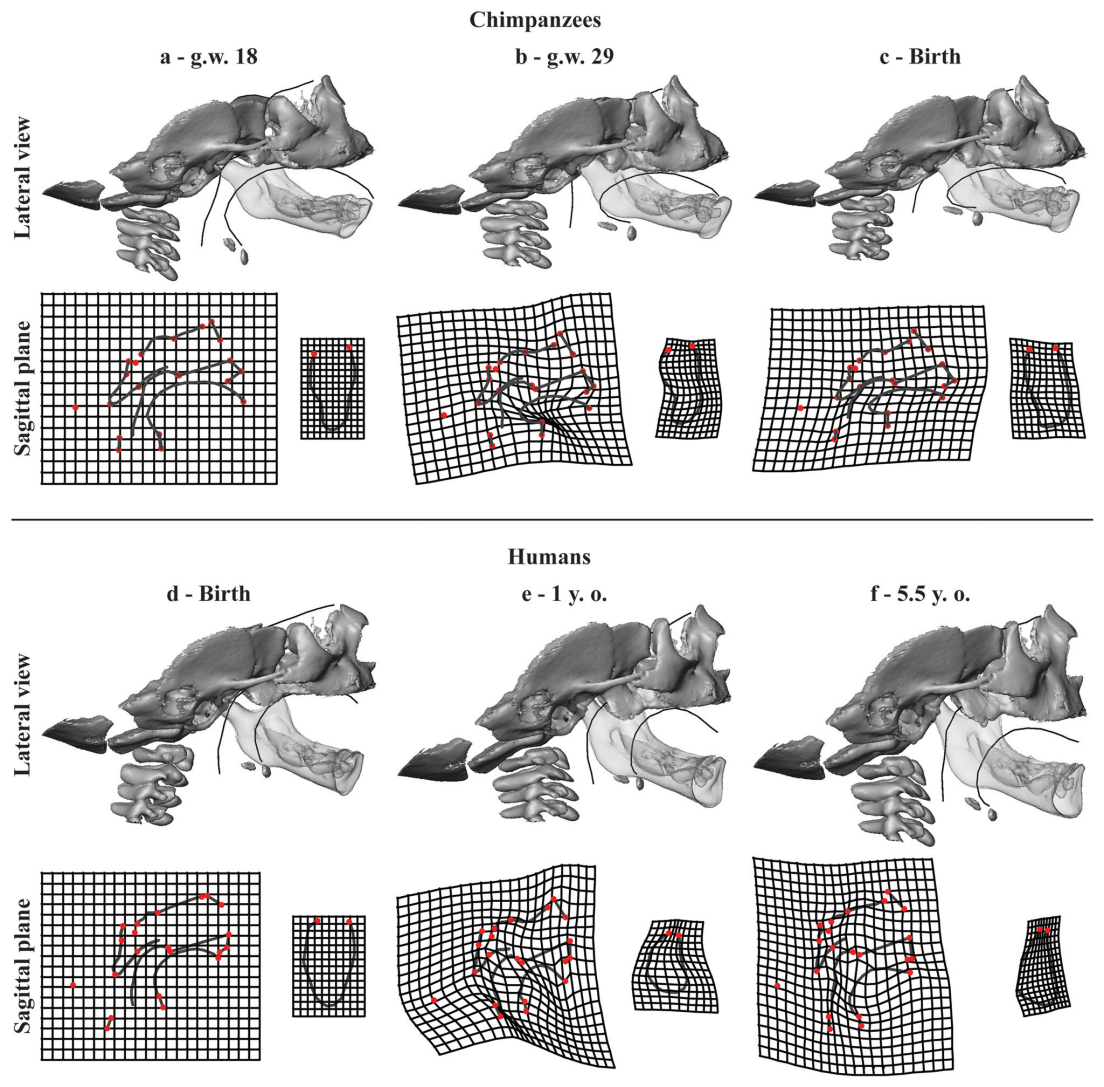
Ontogenetic shape changes. Lateral view: shape changes along the ontogenetic trajectories of chimpanzees (a-c) and humans (d-f) seen in Figure 1. The 3D morphs are regression estimates scaled to unit centroid size in order to focus on the shape changes. The mandible (translucent) was brought into the space of the cervico-craniofacial complex in order to visualize simultaneous shape changes of the tongue and the symphyseal midline. Sagittal plane: the thin plate spline deformation grids illustrate the shape changes of the sagittal structures of block 1 (left) and the symphysis of block 2 (right) from each age stage to its next older stage (exaggerated by factor of 1.5). Red dots: landmarks (listed in Table S1, Figure S1a). Similar shape changes in chimpanzees (a-b) and humans (d-e) simultaneously include: 1) the forward projection of the mental region; 2) the relative horizontal reduction of the tongue and that of the oral cavity; 3) the cranial base flexion; 4) the relative shortening of the anterior cranial base; 5) the clockwise rotation of the posterior region of the upper mid-face; and 6) the forward positioning of the cervical column and the hyoid bone. Growth divergence in chimpanzees (b-c) and in humans (e-f). In humans: 1) vertical growth of the upper mid-face; 2) the rotation of the ramus towards the corpus; 3) lowering of the base of the tongue and the hyoid bone; 4) and the relative anteroposterior narrowing of the pharynx. In chimpanzees: 1) the inferior transverse torus starts to grow posteriorly; 2) the cranial base retroflexes; 3) the upper mid-face rotates forwards; and 4) the cervical column, the hyoid bone and lower part of the pharynx displace backwards.

We carried out a Principal Component Analysis (PCA) of the matrix of shape coordinates augmented by a column of the natural logarithm of Centroid Size (LnCS) – corresponding to a PCA in form space [50] – on the pooled sample to explore the ontogenetic trajectories of blocks 1 and 2 separately (Figure 2). The PCA plots are supplemented by the human and chimpanzee ontogenetic trajectories computed via a piecewise linear regression model of the principal component scores on LnCS.

Afterwards, a sequence of surface morphs was reconstructed to visualize allometric shape variation in both species at the specific points of the ontogenetic trajectories showed in the PCA plots. We first estimated several sets of blocks 1 and 2 via piecewise linear regression of the Procrustes shape coordinates on LnCS. Then the block 1 and 2 surfaces corresponding to the regression estimates were computed using the triangulated surface of one individual and the TPS as an interpolation function [51].

**Figure 3:**
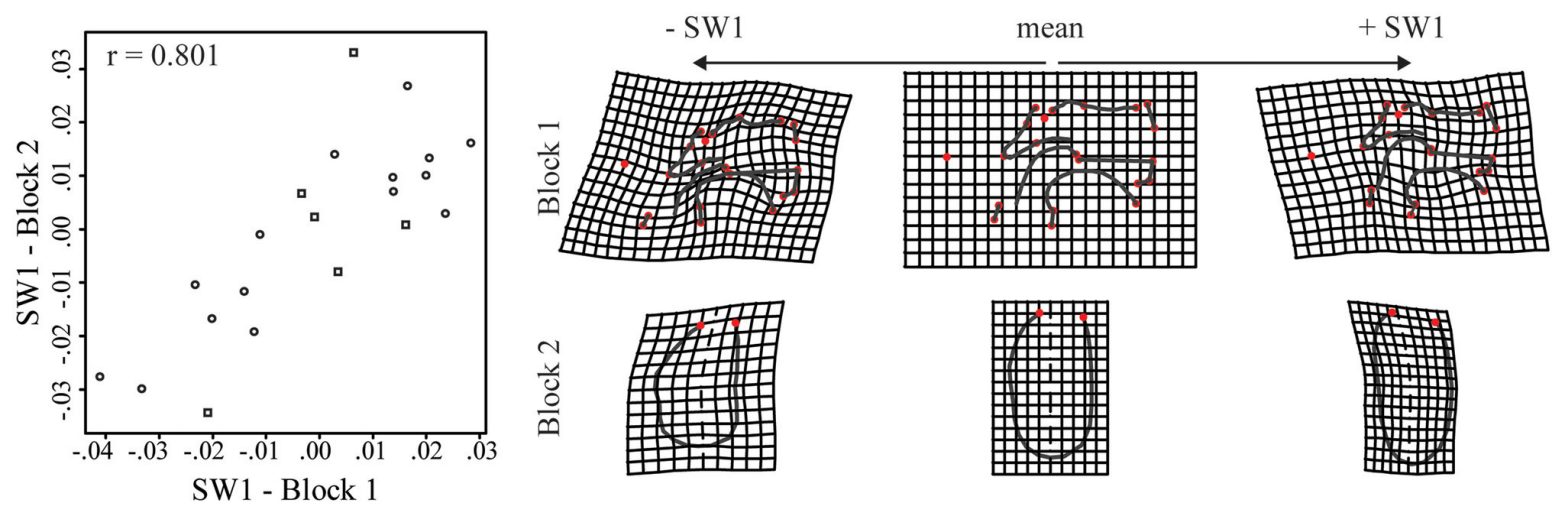
Partial least square analysis. Plot of the first pair of singular warps (SW1s), accounting for 21.92% of the summed squared covariances between these Procrustes coordinates (p-value=0.005). Humans (dots) and chimpanzees (squares) share the same pattern of covariation between the symphysis and the space at the back of the vocal tract. The thin plate spine deformation grids (exaggerated by factor of 2) show the pattern of independent growth. From the Procrustes mean shape of the pooled sample towards the negative SW1 scores, a convex symphyseal middle axis (mental prominence) correlates with a narrowed pharynx, a relatively short anterior cranial base, a backward upper mid-face and a globular tongue. Conversely, towards the positive SW1 scores, a concave symphyseal middle axis is associated with a broad pharynx, a relatively long anterior cranial base, a forward upper mid-face, and a flatter tongue. r: Pearson product-moment correlation coefficient between the first pair of singular warps.

The regression estimates visualize the average association between block 1 and block 2 across the age stages in the sample. But this association does not necessarily imply a causal relationship, i.e. actual developmental integration. We thus use two-block Partial Least Square (PLS) analysis to study integration. As growth is substantial in each subsample, the covariance among morphological units will tend to be very high due in large part to that joint “dependence” [51]. Therefore growth must be removed because it affects all the developmental units under study; otherwise the observed correlation is unreliable [52]. After regressing out size (lnCS) from the shape coordinates in each subsample to remove allometric shape variation and variation in developmental timing [52,53], we carried out PLS analysis on the pooled within-species cross-block covariance matrix [28] between blocks 1 and 2 (semi)landmarks (Figure 3). We assessed the strength of the developmental association between the first pair of singular warps (SW1s) corresponding to the Procrustes variables of blocks 1 and 2 via Pearson product-moment correlation coefficient (*r*). For the significance of the PLS test, the original labels of the second block of Procrustes coordinates are permuted and the *r* between the SW1s is calculated. This is done 1,000 times. The proportion of *r* from the permuted PLS that is equal to or larger than the *r* from the original PLS is equal to the significance level [54].

## Results

For each block, Figure 1 illustrates the human and chimpanzee ontogenetic trajectories in two-dimensional projections of the first three principal components (PCs) of Procrustes form space. Chimpanzees and humans have distinct ontogenetic trajectories. Yet from position *a* (g.w. 18) to *b* (~ g.w. 29) in chimpanzee fetuses and from position *d* (birth) to *e* (~ 1 y.o.) in humans, the trajectories are very similar, indicating similar developmental pathways between the two species. 

Figure 2 illustrates the shape changes of the two blocks along the human and chimpanzee ontogenetic trajectories (Figure 1) and the Movie S1 and Movie S2 offer a comprehensive visualization of the simultaneous shape changes of block 1 and 2 in chimpanzees and humans. 

In chimpanzees from g.w. 18 to approximately g.w. 29 (Figure 2a-b, Movie S1) and humans from birth to about 1 y.o. (Figure 2d-e, Movie S2), the forward projection of the mental region is coordinated with the horizontal reduction of the size of the tongue relative to the size of the oral cavity. This occurs when the anterior cranial base shortens anteroposteriorly and the upper mid-face rotates while the cervical column and the hyoid bone move forwards.

Thereafter, growth starts to diverge between the two species. In humans from 1 y. o. to 5.5 y. o. (Figure 2e-f, Movie S1), the mental region becomes even more prominent along with the lowering of the base of the tongue and hyoid bone and the discrepancy between vertical growth of the upper mid-face and horizontal growth of the pharynx. In chimpanzees from g.w. 29 to birth (Figure 2b-c, Movie S2), the symphysis still remains vertical but at the lingual side of the mental region the inferior transverse torus starts to grow posteriorly. This occurs when the face begins to rotate forwards while the cervical column, the hyoid bone and the lower part of the pharynx displace backwards.

In modern humans after 1 y.o. (Figure 2e-f, Movie S1), the symphyseal midline becomes tear-drop shaped, with a relatively narrow incisor alveolar bone and a broad mental region [55]. The labial side is characterized by a well distinguished *incurvatio mandibularis* [56] – a curve posteriorly convex below the incisor alveolar border. The lingual side also has a posteriorly convex profile which, at its maximum, is the tongue insertion site.

The Figure S2 provides complementary measurements, such as the tongue perimeter and few characteristic angles of the cervico-craniofacial skeleton, and shows that chimpanzees from g.w. 18 to g.w. 29 and modern humans from birth to approximately 1 y. o. have similar trends. The sagittal perimeter of the tongue increases while the cranio-cervical angle and the pharynx aperture angle decrease. These two angles indicate respectively that the cervical column displaces forwards while the face rotates posteriorly. In chimpanzees from g.w. 29 to birth, these angles increase again, in contrast to modern humans. The relative anteroposterior narrowing of the pharynx observed in humans from birth to 5.5 y. o. (Figure S2, Movie S1) is associated with the absence of growth of the lower part of the oropharynx. In addition, when the symphyseal midline becomes tear-drop shaped after the first year of life, the correlation between mandibular growth and tongue growth drops from 0.70 to 0.50, indicating a loss of relative-size synchronicity of the two units.

The PLS analysis was carried out on the 3D Procrustes coordinates of blocks 1 and 2 of a subsample composed of infant humans from birth to 1 y. o. and chimpanzee fetuses prior to birth because they show equivalent shape variations. The plot (Figure 3) of the first pair of singular warps (SW1), shows that humans and chimpanzees have the same pattern of covariation between the two blocks of shape variables, (independent of the average growth pattern which is regressed out of the data). In particular, a convex symphyseal middle axis (mental prominence) correlates with a narrowed pharynx, a relatively short anterior cranial base, a posteriorly oriented upper mid-face and a globular tongue. Therefore, our hypothesis is validated.

## Discussion

Our study demonstrates that development of a vertical symphysis with a mental protuberance in chimpanzee fetuses, and the development of a prominent mental region in humans from birth to approximately 1 y.o. results from a common developmental pathway, related to the shape changes of the vocal tract and the associated arrangement of the tongue and the hyoid bone. In both species, the relative horizontal reduction of the oral cavity originates from the simultaneous forward positioning of the cervical column and the hyoid bone, the backward positioning of the upper mid-face, and the shortening of the anterior cranial base (Figure 2, Movie S1, Movie S2). The correlation between the forward positioning of the mental region, a narrowed pharynx and a globular tongue demonstrates that the reshaping of the symphysis responds to spatial adjustments and constraints at the back of the vocal tract.

In chimpanzee fetuses, the forward positioning of the cervical column and hyoid bone is a consequence of the head flexion towards the throat related to fetal positioning within the womb. In modern humans there is a similar displacement of the cervical column and hyoid bone but it is related to the development of the upright posture [57]. In both species, the backward positioning of the upper mid-face results from the flexion of the cranial base [20,58], and the relative reduction of the horizontal dimension of the oral cavity along with that of the upper-midface and the anterior cranial base, as they are growth counterparts [16]. Therefore, the pharynx, the mandible, the hyoid bone and the tongue are tightly packed together by the crossroads of the development of the upper mid-face and the cervical column. This is illustrated well by the dramatic reshaping of the tongue and the repositioning of the hyoid bone towards the anterior region of the oral cavity.

Our results are the first to provide strong evidence that the mental prominence of modern humans is a by-product of space constraints at the back of the vocal tract, via the reshaping of the tongue which is coping with the retraction of the face and the development of upright posture [16,22]. The tear-drop shaped symphyseal midline characterizing modern humans [55] forms as soon as the base of the tongue and the hyoid bone descent down the throat, and coincides with the loss of synchronicity between mandibular growth and tongue growth. In a previous work, we showed that the tear-drop shape appears when the geniohyoid and the anterior digastric muscle insertions displace downwards and forwards, away from the tongue insertion. The relative relocation of the muscle insertions contributes to the sharply convex lingual profile characteristic of the tear-drop shape of the symphysis [32]. The reshaping of the tongue modifies the position of the suprahyoid muscle insertions as well as that of the hyoid bone relative to the lingual side of the mental region so that the orientation of the suprahyoid muscle force alters the direction of bone growth [59,60]. This seems to coincide in time with the establishment of bone reversal remodelling at the labial side of the symphysis [61], increasing dramatically the mental prominence with the formation of the *incurvatio mandibularis*.

Furthermore, our study demonstrates that during the early postnatal ontogeny of humans, the descent of the tongue and the hyoid bone down the throat, accompanying the descent of the larynx, is tightly linked to the contrasting forces of horizontal and vertical growth of the vocal tract. The large size increase of the tongue cannot fit within the horizontal dimension of the vocal tract. Our results show that the horizontal growth of the lower part of the oropharyngeal space is constrained by growth and displacement of the units surrounding it. Driven by its large size increase and keeping pace with vertical growth of the human retracted face, the descent of the base of the tongue down the throat is to avoid obstruction and maintain the pharyngeal and laryngeal abilities of breathing and swallowing rather than for the needs of speech [18,20,62]. Thus, as for the prominence of the mental region, the large range of acoustically differentiable sounds allowed by a low larynx and the mechanic properties of a tongue with a low posterior base [63], appear to be a by-product derived from the spatial arrangement at the back of the vocal tract compelled by the facial retraction and the development of upright posture.

In addition, our results on developmental integration of the vocal tract in chimpanzee fetuses and infant humans supplement previous studies on African apes and modern humans, implying that the effects of common factors are relatively conserved among hominoids [28-30]. The shared developmental integration of the vocal tract in humans and chimpanzees constitute a strong argument that the different sorts of protrusion of the mental region observed in various extinct hominids could have emerged from a common developmental pathway responding to adjustments and constraints from the space at the back of vocal tract, in a similar developmentally mechanistic way. Therefore, we suggest that the Neanderthal and Aterpuerca specimens, who exhibit a somewhat prominent mental region and who cluster within or close to the range of human variation with respect to their mandibular shape [12], have experienced an equivalent space adjustment at the back of the vocal tract during their ontogeny.

The inverted T-relief on the labial side of the mental region has been proposed as a taxonomic alternative to define the symphysis of modern humans [64]. This morphological feature appears during early fetal life but the craniofacial context and factors linked with its development remain unclear. During early postnatal life, this feature becomes smoother and thicker as the mental region project forwards [31,64]. This suggests that the inverted T-relief may be associated with the same factors as those associated with the prominence; however this needs further exploration.

## Supporting Information

Figure S1 **Templates of 3D (semi)landmarks.** a) Cervico-craniofacial template of 271 landmarks and semilandmarks forming the block 1 of shape variables. b) Mandibular template of 415 landmarks and semilandmarks forming the block 2 of shape variables. Red dots: landmarks; blue dots: curve semilandmarks; green dots: surface semilandmarks. List of landmarks and curve semilandmarks listed in Table S1.
(TIF)

Figure S2 **Absolute measurements.** a) the tongue sagittal perimeter; b) the cranial base angle, measured via the landmarks Basion, Sellae and Foramen Caecum; c) the cranio-cervical angle, measured via the landmarks on Cervical Vertebrae 2, Sellae, Foramen Caecum ; d) the pharynx aperture measured via the angle formed by Cervical Vertebrae 2, Sellae, Posterior Nasal Spine ; and e) the oropharynx length measured between the posterior wall of the pharynx and the epiglottis. In the plots from a to d: the natural logarithm of Centroid Size of the mandible is used as proxy for age to combine humans and chimpanzees in the same plot. In plot e, diamonds: mean value for each age groups. Landmark description in Table S1.
(TIF)

Table S1 **List of landmarks and curve semilandmarks shown in Figure S1.**
(DOC)

Movie S1 **Chimpanzee ontogenetic shape changes.** Interpolations between the regression estimates presented in Figure 2, from the 18^th^ gestational week to birth.
(MPG)

Movie S2 **Humans ontogenetic shape changes.** Interpolations between the regression estimates presented in Figure 2, from the birth week to approximately 5 years old.
(MPG)
