# Supplementary figures and images for: Short Faces, Big Tongues: Developmental Origin of the Human Chin
Source: PLoS One. 2013 Nov 15;8(11):e81287. doi: 10.1371/journal.pone.0081287 (PMC3829973; doi:10.1371/journal.pone.0081287)

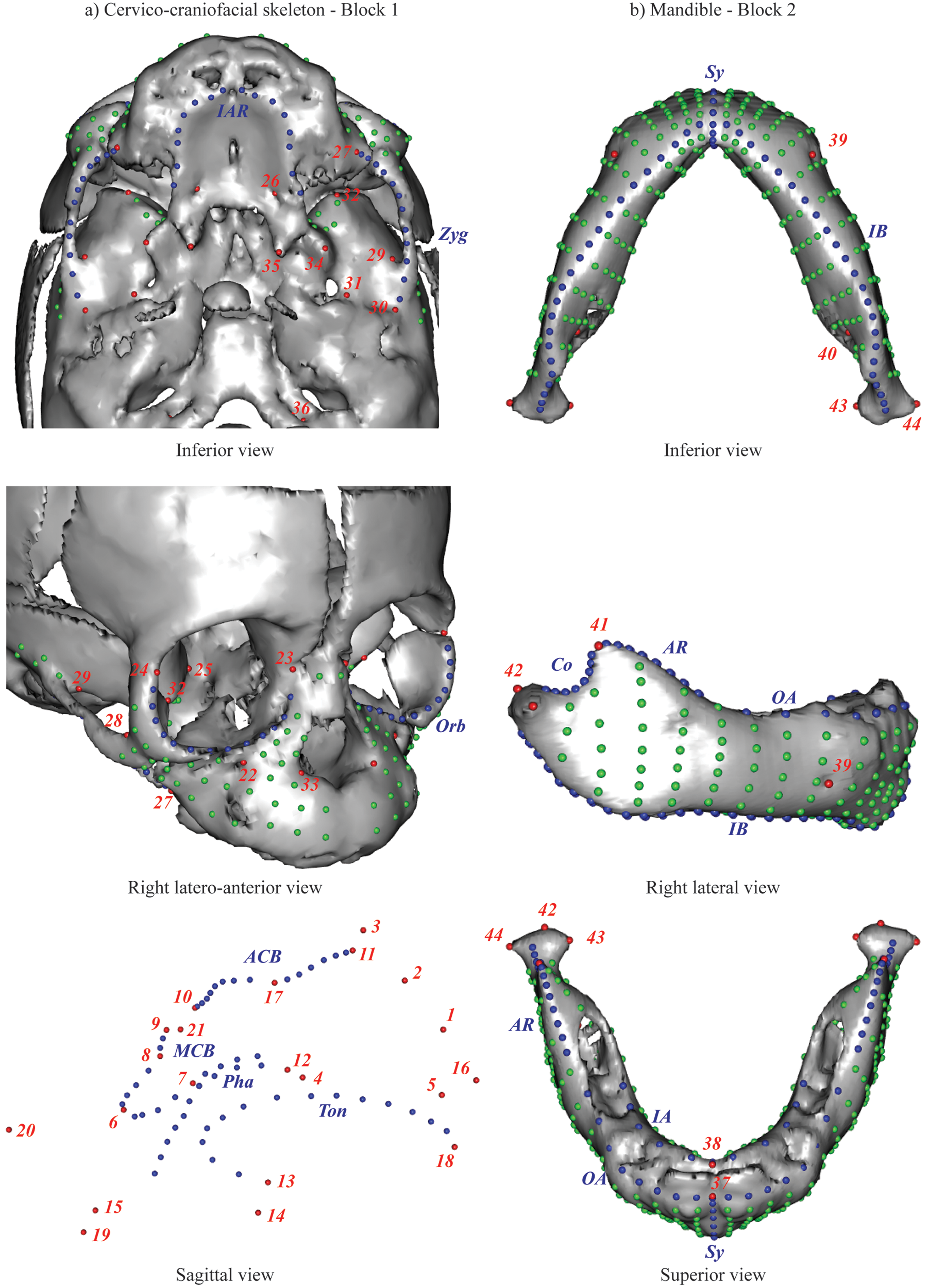

Supplement: Figure S1 — Templates of 3D (semi)landmarks. a) Cervico-craniofacial template of 271 landmarks and semilandmarks forming the block 1 of shape variables. b) Mandibular template of 415 landmarks and semilandmarks forming the block 2 of shape variables. Red dots: landmarks; blue dots: curve semilandmarks; green dots: surface semilandmarks. List of landmarks and curve semilandmarks listed in Table S1. (TIF) [file pone.0081287.s001.tif]

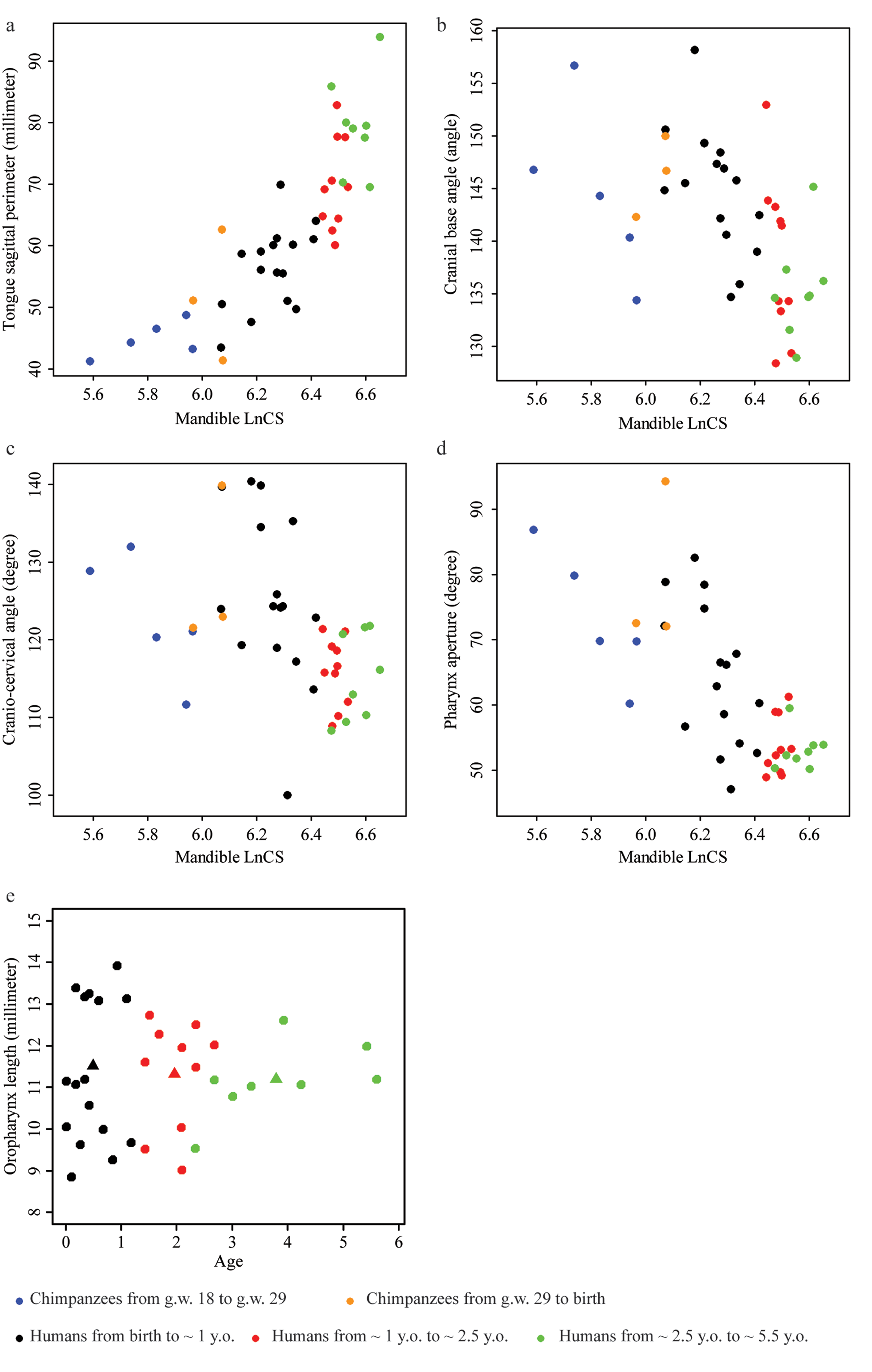

Supplement: Figure S2 — Absolute measurements. a) the tongue sagittal perimeter; b) the cranial base angle, measured via the landmarks Basion, Sellae and Foramen Caecum; c) the cranio-cervical angle, measured via the landmarks on Cervical Vertebrae 2, Sellae, Foramen Caecum ; d) the pharynx aperture measured via the angle formed by Cervical Vertebrae 2, Sellae, Posterior Nasal Spine ; and e) the oropharynx length measured between the posterior wall of the pharynx and the epiglottis. In the plots from a to d: the natural logarithm of Centroid Size of the mandible is used as proxy for age to combine humans and chimpanzees in the same plot. In plot e, diamonds: mean value for each age groups. Landmark description in Table S1. (TIF) [file pone.0081287.s002.tif]
