# Supplementary material for: Short Faces, Big Tongues: Developmental Origin of the Human Chin
Source: PLoS One. 2013 Nov 15;8(11):e81287. doi: 10.1371/journal.pone.0081287 (PMC3829973; doi:10.1371/journal.pone.0081287)
Supplement: Table S1 — List of landmarks and curve semilandmarks shown in Figure S1. (DOC) [file pone.0081287.s003.doc]

**Table S1.** List of landmarks and curve semilandmarks shown in Figure S1

| **Landmarks and curve semilandamarks** | | **Impair / Pair** | **Figure S1** |
| --- | --- | --- | --- |
| *Landmarks cervico-craniofacial skeleton* | |  |  |
|  | Anterior nasal spine | *I* | 1 |
|  | Inferior point of the nasal bone | *I* | 2 |
|  | Superior point of the nasal bone | *I* | 3 |
|  | Posterior nasal spine | *I* | 4 |
|  | Inner alveolar ridge cut by the sagittal plane | *I* | 5 |
|  | Basion | *I* | 6 |
|  | Extracranial point of the spheno-occipital synchondrosis | *I* | 7 |
|  | Endocranial point of the spheno-occipital synchondrosis | *I* | 8 |
|  | Most superior point of the dorsum sellae | *I* | 9 |
|  | Most posterior point of the anterior cranial base | *I* | 10 |
|  | Foramen caecum | *I* | 11 |
|  | Most anterior point of the pharyngeal roof (soft tissue) | *I* | 12 |
|  | Most superior point of the hyoid corpus | *I* | 13 |
|  | Most inferior point of the hyoid corpus | *I* | 14 |
|  | Most anterior and inferior point of the corpus of the cervical vertebra 2 | *I* | 15 |
|  | Outer maxillary ridge cut by the sagittal plane | *I* | 16 |
|  | Superior point of the spheno-ethmoidal synchondrosis | *I* | 17 |
|  | Tip of the tongue | *I* | 18 |
|  | Most anterior and inferior point of the corpus of the cervical vertebra 3 | *I* | 19 |
|  | Opisthion | *I* | 20 |
|  | Deeper point of the sphenoidal sella | *I* | 21 |
|  | Maxillar foramina | *P* | 22 |
|  | Frontal process of the maxilla | *P* | 23 |
|  | Ectoconchion | *P* | 24 |
|  | Rotundum foramina (extracranial) | *P* | 25 |
|  | Grand palatin foramina | *P* | 26 |
|  | Ectomolare | *P* | 27 |
|  | Maximum curvature of the posterior ridge of the zygomatic bone | *P* | 28 |
|  | Anterio-medial junction between the temporal squama and zygomatic arch | *P* | 29 |
|  | Posterior point of the inferior ridge of the zygomatic arch | *P* | 30 |
|  | Medial extremity of the glenoid fossa | *P* | 31 |
|  | Most lateral and inferior point of the orbital surface on the greater wing | *P* | 32 |
|  | Most lateral point of the nasal aperture | *P* | 33 |
|  | Lateral pterygoidal plate | *P* | 34 |
|  | Medial pterygoidal plate | *P* | 35 |
|  | Hypoglossal canal | *P* | 36 |
|  |  |  |  |
| *Landmarks mandible* | |  |  |
|  | Infradentale | *I* | 37 |
|  | Linguale | *I* | 38 |
|  | Mental foramen | *P* | 39 |
|  | Mandibular foramen | *P* | 40 |
|  | Tip of the coronoid | *P* | 41 |
|  | Top of the condyle | *P* | 42 |
|  | Medial extremity of the condyle | *P* | 43 |
|  | Lateral extremity of the condyle | *P* | 44 |
|  |  |  |  |
| *Curve semilandmarks cervico-craniofacial skeleton* | |  |  |
|  | Anterior Cranial Base | *I* | ACB |
|  | Middle Cranial Base | *I* | MCB |
|  | Pharynx | *I* | Pha |
|  | Tongue | *I* | Ton |
|  | Inner Alveolar Ridge | *I* | IAR |
|  | Zygomatic arch | *P* | Zyg |
|  | Orbit | *P* | Orb |
|  |  |  |  |
| *Curve semilandmarks mandible* | |  |  |
|  | Midsymphysis | *I* | Sy |
|  | Outer alveoalar ridge | *I* | OA |
|  | Inner alveoalar ridge | *I* | IA |
|  | Inferior border | *I* | IB |
|  | Anterior ramus | *P* | AR |
|  | Coronoid | *P* | Co |
